# The care for the persistent family aggressor in the perception of nursing students

**DOI:** 10.1590/1518-8345.3991.3287

**Published:** 2020-09-07

**Authors:** Hugo Fernandes, Maykon Brito Brandão, Roberto Alvarenga de Castilho-Júnior, Paula Hino, Conceição Vieira da Silva Ohara

**Affiliations:** 1 Universidade Federal de São Paulo, Escola Paulista de Enfermagem, São Paulo, SP, Brazil.; 2 Scholarship holder at the Conselho Nacional de Desenvolvimento Científico e Tecnológico (CNPq), Brazil.

**Keywords:** Violence, Family Health, Aggression, Nursing, Public Health, Students, Nursing, Violência, Saúde da Família, Agressão, Enfermagem, Saúde Pública, Estudantes de Enfermagem, Violencia, Salud de la Familia, Agresión, Enfermería, Salud Pública, Estudiantes de Enfermería

## Abstract

**Objective:**

to analyze the perception of nursing students about the care given to the persistent family aggressor.

**Method:**

a descriptive study, with a qualitative approach. Madeleine Leininger’s Theory of Diversity and Universality of Care was used as a theoretical reference. The data collection took place with 37 in the last year of at a public university in Southeast Brazil. We used the technique of Projective Design with a Topic and semi-structured interview. The data were analyzed with the content analysis.

**Results:**

the examination of the narratives underpinned the construction of four categories: The care of the aggressor with the family, Recognition of family values and culture, The search for knowledge to care for the aggressor and family, Multidisciplinary and intersectoral care to confront violence.

**Conclusion:**

there is an acknowledgment that the care for the aggressor should not only be individual but should involve all those affected. For this, multidisciplinary work is important, and the search for knowledge on the subject is essential for culturally significant care.

## Abstract (pt)

**Objetivo:**

analisar a percepção de estudantes de enfermagem sobre o cuidado dispensado ao agressor familiar persistente.

**Método:**

estudo descritivo, de abordagem qualitativa. Usou-se da Teoria da Diversidade e Universalidade do Cuidado de Madeleine Leininger como referencial teórico. A coleta foi realizada com 37 concluintes de enfermagem de uma universidade pública da região Sudeste do Brasil. Utilizou-se da técnica do Desenho Projetivo com tema e entrevista semiestruturada. Os dados foram analisados com a análise de conteúdo.

**Resultados:**

o exame das narrativas embasou a construção de quatro categorias: “O cuidado do agressor junto à família”, “Reconhecimento dos valores e cultura da família”, “A busca de conhecimento para cuidar do agressor e família”, “O cuidado multidisciplinar e intersetorial para o enfrentamento da violência”.

**Conclusão:**

há o reconhecimento de que o cuidado do agressor não deve ser individual apenas, mas envolver todos os afetados. Para isto, o trabalho multidisciplinar mostra-se importante, e a busca de conhecimento sobre o tema é algo essencial para o cuidado culturalmente significativo.

## Abstract (es)

**Objetivo:**

analizar la perspectiva de los estudiantes de enfermería sobre el cuidado que se dispensa al agresor familiar persistente.

**Método:**

estudio descriptivo, de enfoque cualitativo. Como marco teórico, se utilizó la Teoría de la Diversidad y de la Universalidad del Cuidado de Madeleine Leinninger. La recolección de datos se realizó con 37 estudiantes del último año de la carrera de enfermería de una universidad pública del Sudeste de Brasil. Se utilizó la técnica de Diseño Proyectivo con Tema y Entrevista Semiestructurada. Los datos se analizaron a partir del análisis de contenido.

**Resultados:**

el examen de los discursos permitió la construcción de 4 categorías: “El cuidado del agresor junto a su familia”, “Reconocimiento de los valores y de la cultura familiar”, “La búsqueda de conocimiento para cuidar al agresor y a su familia”, “El cuidado multidisciplinario e intersectorial para el enfrentamiento de la violencia”.

**Conclusión:**

se reconoce que el cuidado del agresor no debe ser individual, sino que debe involucrar a todos los afectados. A tal efecto, el trabajo multidisciplinario se mostró importante, y la búsqueda de conocimiento sobre el tema es esencial para el un cuidado culturalmente significativo.

## Introduction

Violence, despite its quite complex nature, can be understood as the use of power (physical, psychic or social) for threat or practice, against others or oneself, resulting in injury, deprivation, trauma, harm, and even death^(^ 1 ^-^ 2 ^)^. Among the types of violence is that of family members, characterized as aggression, negligence or omissive acts, of an intentional nature, committed by individuals with family ties to the victim (natural, civil, social or substitute kinship) with the objective of coercion, offense, advantage, demoralization, oppression, or causing pain and suffering to those whom the aggressor judges inferior^(^ 2 ^-^ 3 ^)^.

The isolated concept of violence may not have a coherent meaning if there are no reflections from the perspective of the society that produces it, because there is direct influence of economic, political, social, religious and cultural aspects on daily relationships and peace constructs^(^ 3 ^-^ 5 ^)^. Thus, family violence should be treated as a broad phenomenon, triggered by multiple factors and should be seen in the context, social environment and historical moment that happens, including the perceptions of the individuals involved (victims, aggressors, and witnesses)^(^ 4 ^-^ 5 ^)^.

Among rites, myths and secrets, families can experience violence in a variety of ways, such as sexual violence, gender violence, psychological/moral violence, financial/economic violence, neglect, abandonment, torture, physical aggression, exploitation, harassment, among others^(^ 2 ^-^ 5 ^)^.

Nurses involved in the issue tend to sympathize with the victim, providing zealous assistance and adopting the appropriate measures recommended in flows or lines of care to the person in a violence situation^(^ 5 ^-^ 6 ^)^. However, aggressors tend to be rejected and may even be poorly assisted by professionals, as a movement of reproducing common sense behavior, impulsive and with predefined judgment, especially in cases of frequent offenders, that is, those who practice violent acts eventually or often^(^ 4 ^-^ 6 ^)^.

Recent studies^(^ 7 ^-^ 8 ^)^ indicate that caring for only the victim may not be successful, since the offender may perpetuate his or her violent actions if he or she sees them as natural or does not have his or her needs and feelings accepted and cared for. Besides, offenders may not necessarily be safely removed from the victims, as stated by professional recommendations^(^ 6 ^-^ 7 ^)^.

Another relevant point is that there is a scarcity of studies on nursing care for the persistent family aggressor, i.e., the one that constantly harms one or more members of his or her family nucleus. This can lead professionals and students to persist in conditions of distance and prejudice. There is a particular shortage of publications in developing countries^(^ 8 ^)^. 

The issue of family violence is not usually present in the graduation of nurses and may cause the organization of not very resolute or inefficient care practices^(^ 7 ^-^ 9 ^)^. Thus, the authors of this study ask the following question: what are the perceptions of nursing students about the care for the frequent or persistent family aggressor?

So, the objective of this research is to analyze the perception of nursing students about the care given to the persistent family aggressor.

## Method

Descriptive study, of qualitative approach, carried out with students of the final year of nursing in a public university in the Southeast region of Brazil. These final year students were chosen based on their experience for having completed a large part of the subjects of the nursing degree course and for being a few months away from the legal exercise of their profession. Students regularly enrolled in the last year of nursing course were included and excluded those who had dependence in some curricular subject of the course. Of the 54 eligible students, one was on medical leave, five refused to participate and 11 were unable to participate due to time constraints for the interviews. Thus, the sample was composed of 37 participants.

The data collection was carried out in the second semester of 2018, in private and safe places at the university, in three moments. The first moment occurred with the use of the Topic Projective Design technique^(^ 10 ^)^ in which the students expressed their perceptions about the care to the family aggressor with the use of six hydro-colored pens (equal for all) and sheets of A4 printer paper. This technique allows participants to graphically express their unconscious conceptions about different daily phenomena, allowing the search for understandings as if lived or experienced After the drawings were made, the interviwees were asked to write a story on the back of the sheet and to explain the final material to the researcher. The guiding request was: please draw a person who frequently assaults the family they are part of. No words were used that could induce the gender determination, such as male/female, husband/wife, son/daughter, etc. At the second moment, the students were asked to report the nursing care provided to the family aggressor. The third moment was the turning to the participants, asking them to point out their perceptions of the phenomenon. It was used as a guiding question at this time: what do you understand of the writing, the design, and the care of the persistent family abuser? The three moments were held in a single meeting with each participant. 

The collection was performed by the first and last authors of the study, both with training and experience in the techniques used. All moments were recorded in an electronic audio device, and the drawings were photographed with a digital camera. The total time (projective technique and recorded interview) lasted on average 90 minutes. The participants were identified with the letter E, followed by Arabic numerals, according to the order of the interviews. 

Madeleine Leininger’s Theory of Diversity and Universality of Care (TDUC) was used as a theoretical reference for this research. This theory is based on the following precepts: 1) that human beings are creatures that provide attention and interventions, capable of caring for the needs, satisfaction and survival of others under different conditions, but according to their cultures, needs and scenarios; 2) that health is a culturally defined, evaluated and practiced well-being that reflects the ability of groups to perform their daily activities in a culturally satisfactory, universal and diverse manner at the same time, allowing the creation of a 3) world view through which these people interpret the universe, form attitudes and values about their lives and the world around them, contributing to the development of (4) care as a phenomenon related to helping behavior, support for an individual or group, whose ultimate object would be the balance of a human condition or life^(^ 11 ^-^ 12 ^)^.

In order to analyze the interviews, the content analysis steps developed by Bardin were followed, which were pre-analysis, exploration of the material and processing of the results^(^ 13 ^)^. In the pre-analysis, two authors read, line by line, the transcribed material from the interviews with the students and they search for the elements that could compose the analysis *corpus*. In the exploration of the material, we sought the definition of the categories by classifying the constituent elements in analog groups by frequency of the units of record. There was only one divergence which was analysed by a third researcher, who independently scored his understanding. Finally, the data were inferred and interpreted.

The development of research met national and international standards of ethics in research with human beings and was approved by the Research Ethics Committee of the Federal University of São Paulo, with Opinion No. 2,697,442. The Consolidated Criteria for Reporting Qualitative Research (COREQ) guide was applied to verify the scientific quality of the research.

## Results

Most of the interviewees were female (n=33), between 21 and 32 years old (n=24), single (n=36) and white (n=22).

Regarding the drawings, it was evidenced that all families were heterosexual, nuclear, with one or more children, with members drawn with black or dark lines. In five cases none of the characters had a face. In ten cases, the aggressors were presented with facial expressions of sadness, and in five, with expressions of anger. However, in 17 images, aggressors and victims were illustrated with faces of indifference (n=4) or joy (n=13). All presented images of proximity between the subjects, but only six drawings illustrated physical contact between the characters. The absence of images or environmental characteristics, such as objects, furniture or vegetation (n=25), was evident; in the drawings that contained them, knives, bottles, beverage cans, crutches, knocked over furniture, pets and children’s toys were illustrated.

The stories were quite varied, but there were common characteristics. The main one is that the aggressor was male in 34 stories, being reported as fathers, stepfathers, sons or grandsons. The most described forms of violence were physical (n=16), sexual (n=9), psychological (n=6) and financial (n=3), which occurred repeatedly, motivated by previous violent behavior from the aggressor (n=28), precipitated by alcoholism, illicit drug use, and uncontrollable sexual impulse. Detailed descriptions of power relations between aggressor and victim were noted in all the stories, leading to the persistence of violence and the prolonged suffering of victims and witnesses. In most stories (n=24), families concealed the suffering and maintained happy social behavior because they naturalized the aggressor’s insults or did not know how to deal with the situation. Aggressors and family members remained together in 31 stories, maintaining appearances and social norms. In cases where the perpetrator was away, this occurred due to the escape of family members or the death of the perpetrator.

The analysis of the narratives underpinned the construction of four categories: The care for the aggressor with the family, recognition of family values and culture, the search for knowledge to care for the aggressor and family, multidisciplinary and intersectoral care to confront violence.

In the category The care for the aggressor with the family, participants revealed that domestic violence is an intricate situation for nursing, requiring reflection on the care to the aggressor, without ignoring the needs of other family members, recognizing it as a unit of care in which actions on one affect all others: *I think you have to consider the aggressor’s side and welcome not just him or her, but the whole family, and find some way to treat that family* (E16)*; Understand the family dynamics so that all its members also learn how to deal with this situation involving the aggressor* (E13)*; Trying to find a support network of this family, for example, the mother and father of the aggressor, because they can help in this process* (E17). 

The responses also proved to be aggression a little-discussed topic, presenting difficulties in planning actions: (...) *I don’t know how to explain it. It’s an issue I never stopped to think about... You must have something to do for the person and family, but I do not know* (E34). 

In the category Recognition of family values and culture, some respondents were surprised to have created male aggressors, unconsciously emerging cultural aspects related to gender and power. In most of the drawings and/or stories created, men used verbal, physical or psychological aggression to achieve their goals in relation to women, reproducing the idea of female inferiority: (...) *it’s a cultural issue, this superiority. Nowadays there is a lot of talk about changing that, but I think there is still a story telling women are inferior, so, many times the man ends up being the aggressor* (E27). 

Another aspect present in the narratives was the naturalization of violence, resulting from the idea of maintaining the nuclear model as a paradigm of happiness and fulfillment: *People forgive things in the name of family happiness. People keep quiet because they think it’s going to be better for everyone* (E18). The idea of family harmony above all was highlighted by the students as a reflex of religious and cultural aspects transmitted in a trans-generational way, reproduced frequently and explicitly in violence and by current families: *People keep quiet because they think it’s best to preserve the family* (E10). *Many religions collaborate with this because they preach that families should remain united at any cost, even in situations of violence* (E31). Intergenerationality, which is the passing of behavior between the family generations, was pointed out as one of the causes of the persistent family offender’s behavior, who reproduced what they had learned in the past, and it was up to nursing to somehow understand and intervene in this phenomenon: *Maybe he or she is an aggressor because was assaulted. It was something learned from the father or the origin family* (E1).

In the category The search for knowledge to care for the aggressor and family, the narratives revealed that nursing graduates do not feel confident about the care given to the aggressor and the family because of little training received in their academic training. They also reported the need for more knowledge about family evaluation: *How are we going to convince that person to be treated?* (E22)*; I think we can be trained to take a “double” look at it instead of taking the aggressor as just an evil guy* (E5)*; I think a well-trained nurse could handle it better and understand this family dynamic, we know very little about it. I would say that nothing at all* (E13).

From the reports, it was possible to note that the inclusion of didactic activities and training approaching nursing care for the aggressor in undergraduate curriculum units could reduce the insecurity of the future professional in suggesting behaviors or interventions.

The category Multidisciplinary and intersectoral care to confront violence was generated from participants’ statements that nursing alone would not be effective in caring for the family aggressor. There is a need for inter and intra-sectoral actions that reach various dimensions of the lives of those involved. Some situations involving family assaults led a proportion of those interviewed to the conclusion that, due to little training, or in specific cases, nursing is incapable of successfully intervening in the treatment of those who practice family violence.

A multidisciplinary or even judicial team is needed to solve the violence: *I think therapists and psychologists; it would be professionals more focused on their mental health. You need more people together to take care, even outside the health care area* (E14*); There must be a judicial intervention in the case, to turn to someone in the legal department to help us* (E15)*; In some cases, it is even understandable to accept that is not only us who will be able to do this and request social services, support centers and more drastic measures to be able to intervene* (E29).

## Discussion

The phenomenon of intrafamily violence needs to be deepened in several areas of action, including health, which only a few years ago became more active, transcending the curative aspects and entering into measures for health promotion, prevention of the problem and monitoring of cases. This has been especially noticeable in Brazil in the last two decades, with the addition of the topic to the list of aggravated cases of compulsory notification, the creation of lines of care for people in situations of violence, the empowerment of primary health care through the implementation of centers for the prevention of violence in basic units and the presence of the topic in the agendas of health councils and secretariats^(^ 14 ^)^.

Coordinated care actions in a attention network for the victims have been developed at all levels, with notable advances in secondary and tertiary prevention, correcting deviations from normality as early as possible and trying to recover health conditions, reduce disabilities left behind and reintegrate victims and witnesses in the best possible way into society, especially into the family^(^ 14 ^-^ 15 ^)^. Far from it, however, is the care directed to the family aggressor. Almost always hasty judgments are made, condemning the aggressors to a position of carelessness, because they are not understood as vulnerable in this context^(^ 15 ^-^ 16 ^)^.

But the data obtained from the analysis of the material in this study showed that a special look should be taken at the offender, reflecting on the possibility of care together with the family. Studies indicate that the absence of health care for family aggressors can reflect directly on the victims since there is the possibility of persisting the injuries and damage to the family system^(^ 16 ^-^ 18 ^)^. Moreover, the care to the perpetrator alone may not make full sense without caring for the others involved.

The attention and assistance given to the whole family system, including the offender, favor better and more lasting results, preventing aggression from perpetuating. With a systemic approach, nurses can better understand family life habits, beliefs, myths and secrets that may be contributing factors to aggression and provide effective intervention to the family^(^ 18 ^-^ 20 ^)^.

The care of the aggressor with the families has a greater chance of success since it will allow professionals to understand the context, the family dynamics or structures and the stages of development of families experiencing violence. Often the attention focused on the whole, not just the individual, allows us to recognize the limits and potential of each one facing the phenomenon, making use of the families’ functional capacity. Even the extended family should be evaluated and involved in the care, when possible^(^ 18 ^-^ 20 ^)^.

However, we should note that there are some limits to the aggressor’s therapeutic practices with the family, as special situations can occur in each case, such as legal and judicial restrictions, where the presence of the aggressor increases the risks or exposes victims and witnesses to increased conditions of insecurity. 

Thus, the absence of social actors may be necessary in very special cases, when there are high risks for one or more members or when the presence of some element causes conflicts that are difficult to mediate^(^ 16 ^-^ 21 ^)^. To this end, the Family Health Strategy, present in Primary Health Care in Brazil, is a valuable tool for identifying the factors involved in the care success, because the careful family monitoring allows early recognition of favorable or unfavorable actions^(^ 22 ^-^ 23 ^)^. Another possibility pointed out by the authors are the Singular Therapeutic Projects (*Projetos Terapêuticos Singulares*, PTS) with matrix support from multidisciplinary teams^(^ 24 ^)^, as they allow shared diagnoses, the joint definition of plausible goals, division of responsibilities and reassessment of the efficiency of the care provided to the aggressor and the family.

As pointed out in other studies using TDUC^(^ 11 ^-^ 12 ^)^, those surveyed here also realize that recognition of the values and culture of each family is essential for efficient care, even if in the context of violence the relevance of a family’s values and culture seems strange, *a priori* since violence is almost always an emergency. However, the exploration of information about norms, moral precepts or social rules that the family possesses allows exploring paths in a care project.

Since the family nucleus is loaded with subjectivities and beliefs, often what one has as a consensus of a culture of peace may not be logical. When Leininger studies the behavior of families of different ethnic groups, she realizes that in some societies there are power logics that produce mechanisms for the functioning of that nucleus and that make sense to it^(^ 11 ^-^ 12 ^,^ 25 ^)^. One of these aspects is still relevant: sexism in cultures such as Latin, where women are undervalued, seen as fragile and potentially subject to the men’s will, who in turn are violent and power grantees^(^ 26 ^)^.

As in the unconscious of those researched, man is the main perpetrator of family violence, especially sexual and physical violence. A study conducted in the United States of America has shown that such thinking also occurs in jurists in simulated domestic violence scenarios, even affecting decision making and imposing more severe penalties on men than on women^(^ 26 ^)^. But it is up to health professionals to seek to understand what makes a man a potential aggressor, identifying values, beliefs, and behaviors in order to expand the possibility of culturally correct care planning. Studies indicate that men who commit violent acts in their families reproduce models passed down from previous generations. In other words, the harmful act is a trans-generational message that has been understood as correct or assertive. Within this logic, the family aggressor can also form new aggressors^(^ 27 ^)^.

In addition, it is interesting that in care planning nurses reflect that the violent drive is not necessarily a constant in the life of the aggressor. Perhaps their harmful behavior occurs as a final mechanism of conflict resolution, whose otherwise unsuccessful attempts at resolution have caused atrocious impulses^(^ 22 ^-^ 27 ^)^.

Care for the persistent family abuser has presented itself as a challenge for nursing, especially given the few discussions on this topic during professional training. Therefore, the search for knowledge is a necessary practice for everyone who, directly or indirectly, is involved with the topic. 

One of the emerging possibilities for the training of nurses lies in forensic science, which is concerned not only with the preservation of traces, investigation of scenarios where crimes occur, but also with the assistance provided to the aggressor, in the most varied places and situations, from those of deprivation of liberty to within the home^(^ 28 ^-^ 29 ^)^. 

Understanding the worldview proposed by TDUC may become more timely when nurses dealing with the subject seek the specialty cited, as it reduces the distance between the professional and the one who needs the care^(^ 11 ^-^ 12 ^,^ 25 ^)^, here, the aggressor. The approach of nursing to the subject, from the perspective of the TDUC, can provide a more naturalized service to the health-disease context from the perspective of the actors involved, which allows for the creation of links between those who care and those who are cared for. Also, we can promote human and empathetic care, committed to the other in their values, limits, and culture.

Studies on the topic of domestic violence safely state that the search for knowledge by nurses is a necessity for better care for the victims, witnesses, and aggressors. In addition, such studies cite that the qualification of these professionals can be crucial for decision making, referrals, and outcomes, not only clinical but also psychic and social^(^ 14 ^-^ 15 ^,^ 30 ^)^.

However, the care for the aggressor should not be a solitary assignment. The multidisciplinary action allows for different conceptual perceptions of the facts, which allows for enriching discussions and collectively planning interventions. The multiple training courses in joint action collaborate to find risks, limits, potentialities and possible paths to follow^(^ 21 ^-^ 30 ^)^, as evidenced by the participants of this study. Actions to prevent violence and promote a Culture of Peace, such as conflict mediation, restorative practices and the use of non-violent communication are also measures to be taken by the multidisciplinary health team. These actions reduce the incidence of violence, as well as they can generate medium-term reflexes on harmful cultural aspects, such as *machismo* ^(^ 22 ^-^ 24 ^)^.

Complementing care, intersectoral actions allow the articulation of support networks that foster assertive practices^(^ 31 ^)^. Areas such as justice and citizenship, education, public security, social assistance, and even culture can together add up to efforts that lead to direct or indirect improvements in people’s well-being, their levels of social control and resolution of emerging problems. Restorative practices through justice, teaching non-violent forms of communication in teaching environments, rapid action by public security agencies in cases of family violence, conflict mediation through social assistance and improving access to leisure and culture are some examples of actions that have a positive impact on the care that is a provider of well-being^(^ 8 ^-^ 18 ^)^. 

Therefore, the care of the family offender must take into account that it is not only social conditioning and health determinants that must be improved, such as access to services and treatment but multiple factors that influence satisfaction and quality of life. In addition to the preventive measures adopted by these sectors, collaborative action allows for greater agility in problem-solving, as in cases that require for segregation of the aggressor from the victims.

The limitations of this study are the complexity of the subject and the fact that the data collection was conducted only with nursing students from a public school. Studies aimed at the care to the perpetrator of violence are still rare, leading to difficulty in discussing the findings. The collection in other scenarios could expand the findings or allow comparisons, according to the regionalism of students and socio-epidemiological profiles. However, the limitations do not invalidate the information found, as notorious aspects have emerged regarding what is expected of the care given to the persistent family aggressor.

The research brings as a contribution the possibility of reflections on the subject emergence in the perspective of recognizing the difficulties regarding it and arouse interest in the improvement of care and in research and health policies that meet the demands of the offender so that the cycle of family violence can be interrupted; it also contributes by showing that the use of the technique of projective design can arouse understandings on subjects of great complexity, without having to expose those surveyed directly to them. In addition, it shows that the adoption of the TDUC increases the capacity to understand the reality and dynamics of the interactions of a group and its culture.

## Conclusion

The study revealed that the care given to the family aggressor does not appear to be simple. It requires the one who gives the care the recognition of the context and the values of the family, to re-signify cultural aspects that permeate the relationships and that can answer the questions about the mechanisms of violence. Furthermore, this research has shown that care should be given to the perpetrator, but in harmony with the family. That is, for practices to be culturally meaningful, offender, victim, and witness must be cared for as a unit, avoiding prior trials and unilateral practices.

The students affirmed their technical and formative unpreparedness for the exercise of care with security, however, they recognized the need to search for knowledge that has not been offered at college. Moreover, it is inferred that the subject requires a multidisciplinary and intersectoral approach, given the limitations of nursing and other professions in responding alone to complex situations such as the care to the aggressor and their family. This networked care allows cycles of violence to be broken and beneficial results for the aggressor to be achieved, thus seeking to meet the premises of the culture of peace. 

Finally, the authors point out that perhaps the offer of educational activities on the subject in the modality of university extension can contribute to continuing professional formation.

## References

[1] Aakvaag HF, Thoresen S, Wentzel-Larsen T, Dyb G (2017). Adult victimization in female survivors of childhood violence and
abuse the contribution of multiple types of violence. Violence Against Women.

[2] Schek G, Silva MRS, Lacharité C, Bueno MEM (2017). Organization of professional practices against intrafamily
violence against children and adolescents in the institutional context Rev.
Latino-Am. Enfermagem.

[3] Griffith R (2017). Domestic violence protection measures. Br J Nurs.

[4] Shoqirat N, Almajali A, Alsaraireh A (2019). From a family home to hell experiences and consequences of
intimate partner violence among married Jordanian women. Issues Mental Health Nurs.

[5] Clements CM, Bennett VE, Hungerford A, Clauss K, Wait SK (2018). Psychopatology and coping in survivors of intimate partner
violence associations with race and abuse severity. J Aggress Maltreat Trauma.

[6] Rigol-Cuadra A, Galbany-Estragué P, Fuentes-Pumarola C, Burjales-Martí MD, Rodríguez-Martín D, Ballester-Ferrando D (2015). Perception of nursing students about couples' violence knowledge,
beliefs and professional role. Rev. Latino-Am. Enfermagem.

[7] Verdolini N, Murru A, Attademo L, Garinella R, Pacchiarotti I, Bonnin CM (2017). The aggressor at the mirror psychiatric correlates of deliberate
self-harm im male prision inmates. Eur Psychiatry.

[8] Ferreira MNX, Hino P, Taminato M, Fernandes H (2019). Care of perpetrators of repeat family violence an integrative
literature review. Acta Paul Enfermagem.

[9] Maquibar A, Hurtig AK, Cases CV, Estalella I, Goicolea I (2018). Nursing students' discourses on gender-based violence and their
training for a comprehensive healthcare response a qualitative
study. Nurse Educ Today.

[10] Backos A, Samuelson KW (2017). Projective drawings of mothers and children exposed to intimate
partner violence a mixed methods analysis. Art Therapy.

[11] Rohrbach-Viadas C (2015). Historic perspectives from anthropology Reflections proposed to
Transcultural Nursing. Invest Educ Enferm.

[12] Jiménez-Ruiz I, Almansa Martínez P (2017). Female genital mutilation and transcultural nursing adaptation of
Rising Sun Model. Contemp Nurse.

[13] Mendes RM, Miskulin RGS (2017). Content analysis as a methodology. Cad Pesqui.

[14] Garbin CAS, Dias IA, Rovida TA, Garbin AJ (2015). Challenges facing health professionals in the notification of
violence mandatory implementation and follow-up procedures. Ciênc Saúde Colet.

[15] Avanci JQ, Pinto LW, Assis SG (2017). Treatment for cases of violence by Brazilian emergency services
focusing on family relationships and life cycles. Ciênc Saúde Colet.

[16] Fowler DR, Cantos AL (2016). Miller SA Exposure to violence, typology and recidivism in a
probation sample of domestic violence perpetrators. Child Abuse Negl.

[17] Kelly L, Westmorland N (2016). Naming and defining domestic violence lessons from research with
violent men. Fem Rev.

[18] Cantos AL, Kosson DS, Goldstein DA, O'Leary KD (2019). Treatment impact on recidivism of family only vs generally
violent partner violence perpetrators. Int J Clin Health Psychol.

[19] Razera J, Gaspodini IB, Oliveira EL, Neis LF, Falcke D (2018). Couple therapy in contexts of intimate partner violence
integrative literature review. Contextos Clínic.

[20] Madalena M, Carvalho LF, Falcke D (2018). Intimate partner violence the predictive power of experiences in
the family of origin and of personality disorder traits. Trends Psychol.

[21] Llor-Esteban B, García-Jiménez J, Ruiz-Hernández JA, Godoy-Fernández C (2016). Profile of partner aggressors as a function of risk of
recidivism. Int J Clin Health Psychol.

[22] Schimidt B, Coelho ESB (2017). Approach to family violence in the Family Health Strategy review
of literature. Psicol Argum.

[23] Garbin CAS, Rovida TAS, Costa AA, Garbin AJI (2016). Recognition and reporting of violence by professionals of the
Family Health strategy. Arch Health Invest.

[24] Vall B, Päivinen H, Holma J (2017). Results of the Jyväskylä research project on couple therapy for
intimate partner violence topics and strategies in successful therapy
processes. J Fam Ther.

[25] Giger JN (2017). Transcultural nursing, assessment & intervention.

[26] Russell B, Kraus S (2016). Perceptions of partner violence how aggressor gender,
masculinity/femininity, and victim gender influence criminal justice
decisions. Deviant Behav.

[27] Valgardson BA, Schwartz JA (2018). An examination of within and between family influences on the
intergenerational transmission of violence and maltreatment. J Contemp Crim Justice.

[28] Olsson H, Kristiansen LP (2017). Violence risk assessment in clinical practice how forensic nurses
experience violence risk assessment in daily work - a qualitative interview
study. Glob J Health Sci.

[29] Olsson H, Audilv A, Strand S, Kristiansen L (2015). Reducing or increasing violence in forensic care a qualitative
study of inpatient experiences. Arch Psychiatr Nurs.

[30] Linnarsson JR, Benzein E, Årestedt K (2015). Nurses' views of forensic care in emergency departments and their
atitudes, and involvement of family members. J Clin Nurs.

[31] Serrano-Gallardo MP (2019). Intersectorality, key to address Social Health Inequalities Rev.
Latino-Am. Enfermagem.

